# The effectiveness of digital interventions for enhancing empathy in adults: a meta-analysis of randomized controlled trials

**DOI:** 10.1007/s12144-025-08981-8

**Published:** 2026-02-17

**Authors:** Camille Y. Williams, Qiang Xie, Elvan Muratoglu, Simon B. Goldberg

**Affiliations:** 1 Department of Counseling Psychology, University of Wisconsin, Madison, WI USA; 2 Center for Healthy Minds, University of Wisconsin, Madison, WI USA; 3 Michigan Medicine, University of Michigan, Ann Arbor, MI USA

**Keywords:** Empathy, Smartphone, Virtual reality, RCT, Meta-analysis, Systematic review

## Abstract

The current study examined the effectiveness of digital interventions for enhancing empathy in adults. We conducted a meta-analysis of randomized controlled trials (RCTs) of interventions compared with non-specific (i.e., not intended to be therapeutic) and active control conditions (i.e., intended to be therapeutic). Across 24 studies, including 26 comparisons (*n* = 3,137), the overall estimated effect size was statistically significant (*g* = 0.19, 95% CI [0.05, 0.32], *p* = .006). However, when accounting for publication bias using trim-and-fill analysis, the estimated effect size became smaller and non-significant (*g* = 0.07, 95% CI [-0.07, 0.22], *p* = .322). Studies with follow-up data were analyzed separately (*k* = 6), yielding a small effect (*g* = 0.29, 95% CI [0.07,0.5], *p* = .008) which was robust to trim-and-fill analysis. No significant moderators were identified. An exploratory analysis of studies separated by control group type revealed a small positive effect for studies with non-specific control groups (*g* = 0.22, 95% CI [0.12, 0.33], *p* < .001) which was robust to trim-and-fill analysis. The results suggest there is potential for digital interventions to have a positive impact on enhancement of empathy in adults, although digital interventions do not appear to outperform active control conditions. For digital interventions to most fully deliver on their potential, they may require more advanced technology that can offer attuned interaction that mimics a human relationship, as well as offer feedback to participants.

**Supplementary Information:**

The online version contains supplementary material available at 10.1007/s12144-025-08981-8.

Broadly conceptualized as the ability to imagine another’s perspective and subsequently have a vicarious emotional experience, empathy plays a critical role in human interaction (Cuff et al., 2016). It underpins healthy and fulfilling relationships with others and with oneself (De Paul & Guibert, 2008; Gibb & Abela, 2008). Researchers describe empathy as a trait with a developmental trajectory, as a state influenced by specific social contexts (Cuff et al., 2016), and as a behavior or skill that can be learned over time (Ratka, 2018). In addition, empathy can be categorized into cognitive and affective components. Cognitive empathy is the recognition of emotion in another (Gerdes et al., 2010) and can be understood as a top-down process that involves producing a working model of others’ emotional states based on visual, auditory, and context clues and thus requires holding and employing information within the working memory (Decety & Meyer, 2008). In contrast to cognitive empathy, affective empathy is a bottom-up process of experiencing another’s emotions oneself and responding with an appropriate emotion (Cuff et al., 2016). Empathy can also have a behavioral component (i.e., outward expressions of empathy; Overgaauw et al., 2014).

Empathy is important for individuals, relationships, and society. Evidence overwhelmingly suggests that increases in empathy lead to higher quality of life, healthy and positive relationships, and improved well-being (Morelli et al., 2015; Vinayak & Judge, 2018). On a societal level, empathy plays a powerful role in the welfare and progress of all communities and cultures. Indeed, the enhancement of empathy has been a focus of many researchers in developmental and educational psychology because it has the power to shape responsible citizenship and societal change within the next generation (Krznaric, 2014; Rigg & van der Wal-Maris, 2020). Additionally, empathy in helping professionals such as physicians is associated with better outcomes for patients (Hojat et al., 2011). Numerous interventions have been created for fostering empathy in children, such as socio-emotional learning curriculums for preschool and elementary school classrooms (Rosenthal & Gatt, 2010; Schonert-Reichl et al., 2012). While considerable research has examined interventions that target empathy in the early stages of life, relatively less attention has been given to interventions that might enhance empathy in adults.

Interventions that have been studied for enhancing empathy in adults have taken various forms, the most common of which have consisted of in-person trainings that include games, role play, didactics (lectures), demonstrations and practice, interpersonal-process-recall videotape-feedback models, guided meditation, and micro-training (counseling micro-skills trainings; Lam et al., 2011; Teding van Berkhout & Malouff, 2016). Research evaluating these empathy trainings indicate that they are generally effective. Indeed, a meta-analysis conducted by Teding van Berkhout and Malouff (2016) examined the efficacy of empathy training, primarily focusing on traditional, non-digital interventions. The results showed a medium magnitude effect (*g* = 0.63) favoring empathy training over control groups. While it is established that traditional empathy trainings are effective, the efficacy, scalability, and accessibility of these interventions are limited by lack of immersive features and practical barriers such as cost, the need for physical transportation, and limited resource availability.

Digital interventions have the potential to enhance the efficacy, scalability and accessibility of empathy trainings. For the purpose of this meta-analysis, we define digital interventions broadly to encompass a variety of digital technologies including computers, mobile devices, online tools, virtual reality (VR), digital role-playing simulations, and video games, among others that are used with the aim of affecting an outcome or change. Digital interventions may elicit higher engagement and result in more significant and longer-term impact than traditional trainings. For example, in a study that aimed to elicit empathy for the homeless, Herrera et al. (2018) found that participants in the VR perspective-taking task had more positive, longer-lasting attitudes toward the homeless and signed a petition supporting the homeless at a significantly higher rate than participants who completed a traditional perspective-taking task. It is likely that the immersive nature of VR, simulation, video game, and computer game platforms provide a more realistic, embodied experience of empathy (Boltz et al., 2015), potentially resulting in more potent impact. Additionally, these types of digital interventions may be more effective because they offer a space that is safe to make mistakes while developing new skills (Sweigart et al., 2014). Moreover, digital interventions are easily scalable, and many are accessible to anyone with an internet connection (Linardon et al., 2024).

Despite the promise of digitally delivered empathy training, current findings on the efficacy of digital interventions for empathy are mixed. While some studies of digital interventions show encouraging results for improving empathy (e.g., Mueller et al., 2018), some indicate that digital platforms can hinder the impact of an empathy intervention. Porcino et al. (2017), for example, found that VR can cause motion sickness, disorientation, and other discomfort that mitigated the intervention’s effects. Likewise, some video game and simulation interventions indicate that participants end up increasing empathy only for characters with similar identities or backgrounds in comparison to characters of other cultures and identities (Paiva et al., 2005). Some critics also argue that digital interventions are unlikely to be beneficial for enhancing empathy, as digital culture has reduced social interaction, increased individualism, and decreased empathic responses among younger generations (Gorry, 2009; Misra et al., 2016).

## Present study

Given the mixed findings on the efficacy of digital interventions for empathy, the present meta-analysis sought to synthesize the evidence from the available randomized controlled trials (RCTs) testing these interventions. To date, no meta-analysis to our knowledge has synthesized the effects of digital interventions on empathy. This study filled this gap by providing the first meta-analysis of their impact. By integrating mixed findings across studies, the present meta-analysis can clarify the efficacy of these interventions, thereby informing both clinical practice (i.e., whether or not these interventions should be recommended) as well as guiding future research on the development of evidence-based interventions for empathy. We focused specifically on empathy training in adults, given previous research on empathy predominantly focused on early stages of life and less is known about empathy development later in life (Rosenthal & Gatt, 2010; Schonert-Reichl et al., 2012). This is particularly important given that adults are at a different stage of neurocognitive and psychosocial development than children or adolescents, which may influence how they engage with and are impacted by digital empathy interventions. Demonstrating that digital interventions have the capacity to increase empathy in adulthood may have important implications for enhancing individual, relational, and societal health. According to Social Cognitive Theory (Bandura, 1986), learning occurs through the dynamic interaction of personal, behavioral, and environmental factors, where individuals acquire new behaviors by observing and modeling others. Digital platforms that include video modeling, virtual reality, interactive simulations, and feedback to users may provide adults with opportunities to observe empathic behaviors, practice responding in social situations, and receive reinforcement that strengthens empathic skill development. Thus, we hypothesized that digital interventions would have a positive impact on empathy development in adults.

In addition to evaluating the overall impact of digital intervention on empathy in adults, we were also interested in factors that may influence the effectiveness of this approach. We examined the following moderators: neurological or psychological disorder, control group types, career type, type of digital interventions, age, dose, deliberate practice or feedback. Specifically, we hypothesized that digital interventions would have a smaller impact on empathy among individuals with conditions associated with impaired empathic capacity (e.g., autism spectrum disorder, traumatic brain injury, antisocial personality disorder, or schizophrenia), as these conditions may limit responsiveness to interventions. We also expected larger effects when interventions were compared to non-specific control groups (i.e., comparisons not intended to be therapeutic) vs. active control groups (i.e., those intended to be therapeutic; Wampold et al., 1997). We anticipated stronger effects among adults in helping professions vs. other careers, as they may be more motivated to cultivate empathy due to job demands. VR-based interventions were expected to outperform other digital formats, due to their immersive and embodied nature. Younger adults were expected to benefit more than older counterparts, given their greater familiarity with digital technology. Lastly, we predicted that digital interventions with higher dosage or longer duration, and those including deliberate practice and/or feedback, would yield larger effects, as repeated exposure, practice, and feedback might support users in refining empathy skills and consolidating their learning.

## Methods

### Protocol and registration

The current study was preregistered through the Open Science Framework (https://osf.io/b9ukd?view_only=b70bba762eee4c11ac3528ddd3e79b38) and was conducted based on the Preferred Reporting Items for Systematic Reviews and Meta-Analyses (PRISMA) guidelines (Moher et al., 2009).

### Eligibility criteria

The purpose of this meta-analysis was to examine the effectiveness of digital interventions in enhancing empathy in adults. Studies were selected based on the following criteria: (a) RCT, (b) delivered a digital intervention, (c) utilized an empathy outcome measure that included a cognitive, affective, or behavioral component of empathy, or any combination of the three, and (d) included an adult population (i.e., samples ≥ 18 years old, on average). We focused on interventions that were designed to be delivered digitally, such as a VR immersive experience or an interactive smartphone app. Examples of interventions that are digital but were not included in this meta-analysis were video games used purely for entertainment purposes, traditional psychotherapy delivered via telehealth, or a psychoeducational document delivered on an e-reader. The first example is not an intervention, and the latter examples involve a digital component, but the digital aspect of the experience is not central to the intervention’s delivery nor its effectiveness. Of note, if a study examined cognitive empathy only, they needed to explicitly name it as “cognitive empathy” rather than “theory of mind” or other terms. Studies with non-digital control conditions were eligible. When statistics used to calculate effect sizes were not included in the published findings, the original study authors were contacted to provide necessary data. There were no geographic, cultural, or publication date restrictions for study inclusion in this meta-analysis. Studies published in English, Spanish, and German were included.

### Information sources

A systematic literature search was conducted on February 23, 2024 to locate relevant studies that met inclusion criteria. The following databases were searched: PsycINFO, PubMed/Medline, CINAHL Plus with Full Text, Cochrane Trials, Scopus, Web of Science, and ProQuest Dissertations and Theses Global. In addition, we utilized the reference lists of previously published meta-analyses (e.g., Teding van Berkhout & Malouff, 2016) to retrieve additional relevant studies. The study was pre-registered prior to conducting the literature search in order to increase the transparency and rigor of the evaluation.

### Search

The key terms used for the literature search included: (technology OR digital OR virtual OR internet) AND (intervention OR program OR training OR treatment) AND (mentalizing OR “theory of mind” OR “emotion recognition” OR perspective-taking OR “empath*”) AND (“random*”). These search terms were developed by considering the most common words used to describe each factor in our research question, such as terms like “mentalizing,” which is synonymous with cognitive empathy (Cerniglia et al., 2019). A research librarian was also consulted for best practices in developing an exhaustive list of search terms. In order to reduce publication bias (Borenstein et al., 2009), dissertations and unpublished findings were included in the literature search. Databases were searched since inception.

### Study selection

The first author and one additional coder independently coded all studies for inclusion/exclusion. Coder training included an overview of the coding manual followed by an initial phase of coding a small number of titles/abstracts that we then examined together to discuss discrepancies. After all studies were coded by both coders independently, coders reconvened to discuss any discrepancies. Discrepancies were resolved through discussion and consensus, returning to original articles when indicated and consulting with the senior author as needed. Interrater reliabilities for inclusion at title and/or abstract and full text levels were good to excellent (i.e., *ICC* ≥ 0.60; Cicchetti, 1994).

### Data collection process

Standardized spreadsheets were used to record study characteristics and effect size information for each of the trials. Data were independently extracted by the first author and a second coder. Studies were excluded if they were still recruiting participants or if authors did not respond when contacted regarding availability of data.

### Data items

All the studies included in this meta-analysis were intervention studies. Pre- and post-test means and standard deviations from the intervention and control groups were utilized to derive between-group effect sizes. For studies that provided post-test outcome data only (missing baseline data), we compared the groups at post-test. When baseline *n*s were not reported, we used post-test *n*s.

Several continuous and categorical variables were coded for descriptive purposes and for moderator testing. Included studies were coded on characteristics of the study and study design, characteristics of the sample, and characteristics of the intervention. Characteristics of the study and study design included: (a) publication year, (b) method of group assignment, (c) number of groups, (d) type of control group, and (e) empathy measure used. Characteristics of the sample included: (a) mean age, (b) percentage female, (c) percentage racial/ethnic minority, (d) diagnosis (i.e., autism spectrum disorder, schizophrenia, traumatic brain injury, antisocial personality disorder, or none), (e) career type (i.e., whether or not the participants had a career in healthcare or education), (f) stage of career (e.g., trainee vs. non-trainee), and (g) country of origin. Characteristics of the intervention included: (a) group vs. individual delivery, (b) type of digital intervention (i.e., VR, video game, simulation, online module(s), smartphone app, other), (c) whether the intervention was delivered in combination with human support (e.g., smartphone application + in-person exercises), (d) comparison condition or control group type (i.e., active vs. non-specific; Wampold et al., 1997), f) dosage (i.e., hours), and g) whether the intervention included deliberate practice or feedback.

### Summary measures

As the studies included in this meta-analysis were all RCTs of interventions, effect sizes were calculated as Becker’s $\:\varDelta\:$ (1988), defined as the difference between within-group standardized mean differences (i.e., Cohen’s *d*). For studies that did not report Cohen’s *d*, pre- and post-test means and standard deviations were used to calculate it (Cohen, 1988). Becker’s (1988) $\:\varDelta\:\:$accounts for potential baseline differences rather than relying exclusively on between-group differences at post-test. The formulas that were utilized to make these calculations with pre- and post-test means and standard deviations are listed below:

1 $$\:{d}_{within}=\frac{{M}_{pre}-{M}_{pre}}{{SD}_{pooled}}\:$$

2 $$\:var\:\left({d}_{within}\right)=(\frac{1}{n}\:+\frac{{d}^{2}}{2n}\:)\:\times\:\:2(1\:-\:r)$$

If the included studies did not report *r* (the correlation between pre- and post- scores), a correlation of *r*_xx_ = 0.50 was assumed between time points (Hoyt & Del Re, 2018). It is also important to note that the pooled SD in the denominator of the above equation is from pre-treatment (Becker, 1988).

In order to account for the upward bias of Cohen’s *d* (an overestimation of the absolute value of standardized mean difference in smaller samples), *d* values were converted to Hedge’s *g* using correction factor *J* (Borenstein et al., 2009). The between-group effect size for the difference between the digital intervention and control groups was then calculated using Becker’s $\:\varDelta\:$ (1988):

3 $$\:{\Delta\:}={g}_{within-\:}^{T}{g}_{within}^{C}$$

4 $$var\:\left(\triangle\right)=var(g_{within}^T)+var(g_{within}^C)$$

Superscript T refers to the digital intervention and C refers to comparison condition. The Becker’s $\:{\Delta\:}$ formula can be reversed, so it is important to note the order (Treatment – Control) for interpretational implications. In this case, a positive value will favor Treatment, and a negative value favors Control. For each reported effect size in each study included in this meta-analysis, calculations were completed using the ‘metafor’ (Viechtbauer, 2010) and ‘MAd’ packages in R (Del Re & Hoyt, 2014). A random-effects model was used, with effect sizes weighted based on estimates of both within-study and between-study variance. For studies that measured cognitive and affective empathy outcomes separately, we combined them into a single value. Effects at follow up time points were analyzed separately, when available.

### Moderator analysis

We hypothesized that various factors (e.g., neurological or psychological disorder, career type, type of digital intervention, etc.) may influence the impact of digital interventions on empathy in adults. We examined both continuous (e.g., age of participants) or categorical (e.g., type of profession, type of digital intervention) moderators using meta-regression methods implemented in the ‘metafor’ package in R (Viechtbauer, 2010). Continuous moderators included age and treatment length (in weeks). Categorical moderators included presence of a neurological or psychological disorder, control group type, career type, deliberate practice or feedback, and type of digital intervention. These moderators were dummy coded and included as predictors in the meta-regression.

### Synthesis of results

#### Aggregation of effect sizes within studies

As some studies reported multiple effect sizes that corresponded to different measures of empathy, aggregation at the study level was necessary. After calculating Becker’s $\:{\Delta\:}$, as described above, all dependent effect sizes within a given study were aggregated using the ‘MAd’ package (Del Re & Hoyt, 2018), in accordance with Hunter & Schmidt’s (2004) recommendations.

#### Tests of heterogeneity

The variability of the effect sizes was examined using a heterogeneity test to determine if variability is greater than what would be expected by chance. Specifically, we examined the *Q* and *I*^*2*^ statistics. The *Q* statistic was used to test whether the observed between-study variability was greater than would be expected by chance alone (Borenstein et al., 2009). The *I*^*2*^ was calculated to determine what percentage of the observed variance reflects actual differences among studies beyond the effects of random error (Borenstein et al., 2009). The extent of heterogeneity can be interpreted based on Higgins and Thompson’s (2002) guidelines: 25–49% is small, 50–74% is medium, and 75–100% is a large amount of heterogeneity. A significant *Q* or an *I*^*2*^ in the medium, or large range indicates that variation from the true effect size may be due to (measured or unmeasured) moderating variables.

### Assessment of reporting bias

Studies with significant results tend to be published more so than studies with less significant results (Borenstein et al., 2009), which ultimately influences results of meta-analyses if not accounted for with assessment of reporting bias. The most effective way to reduce bias is to perform a comprehensive and exhaustive literature search. In addition, we examined the impact of potential publication bias using the trim-and-fill procedure (Duval and Tweedie, 2000).

Trim-and-fill analysis may be adequate for assessing publication bias, but if a meta-analysis has high heterogeneity, trim-and-fill’s ability to correct for bias may be limited (Carter et al., 2019; Terrin et al., 2003). Moreover, trim-and-fill analyses are based on a fixed effects model, rather than the random effects model we used. Thus, trim-and-fill analyses were interpreted cautiously. Subsequently, visual inspection of a contour-enhanced funnel plot allowed for more thorough assessment of publication bias. Contour lines added to the funnel plot mark conventional milestones in levels of statistical significance and can aid in discerning if studies are missing in areas of statistical non-significance vs. higher significance. Studies missing in areas of statistical non-significance suggests that asymmetry is due to publication bias, whereas studies missing in areas of higher statistical significance may suggest factors other than publication bias, such as variable study quality (Peters et al., 2008).

## Results

### Study selection

**Fig. 1:**
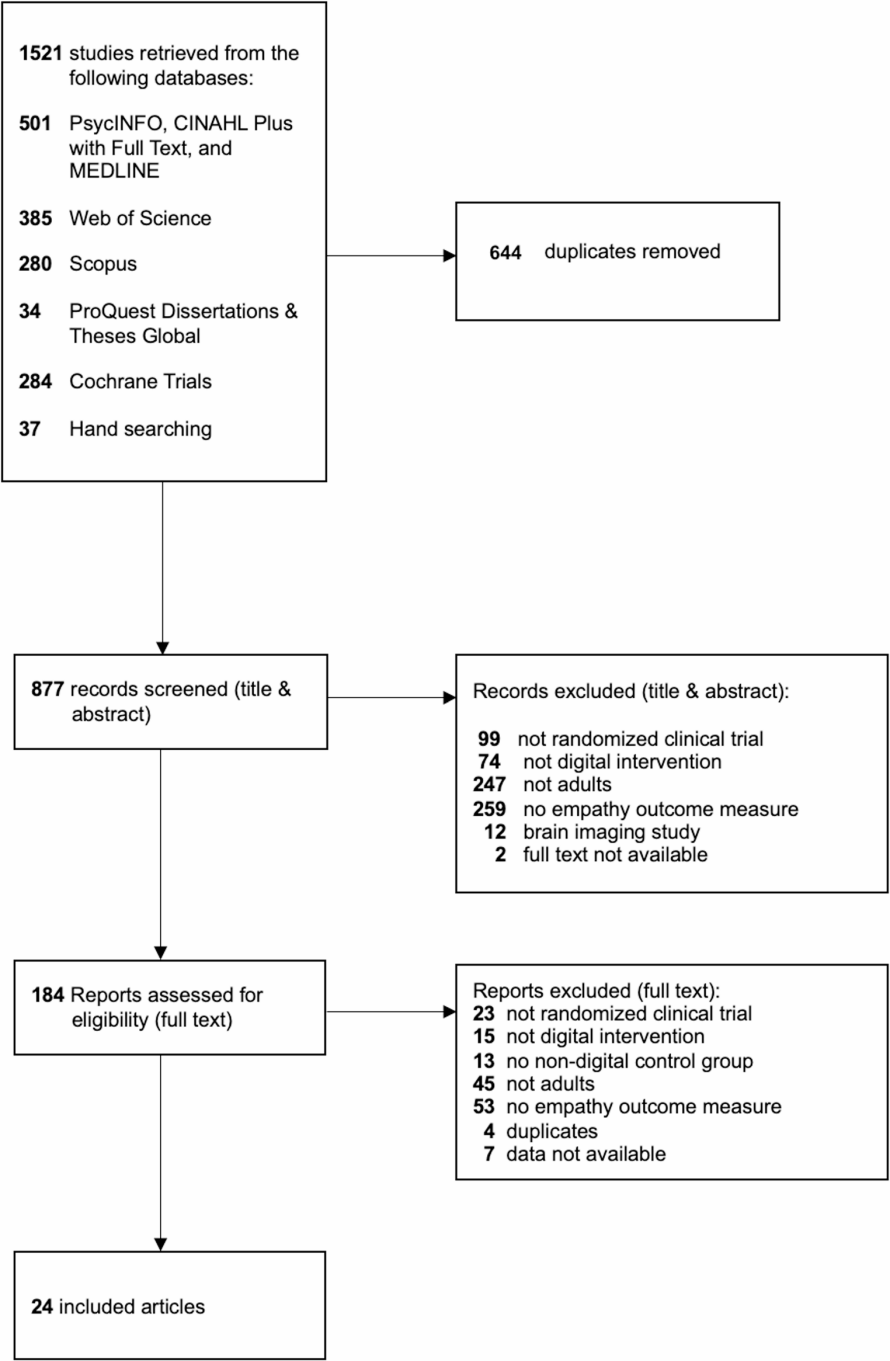
PRISMA Flow Diagram

The search yielded 1521 citations. We removed 644 duplicates and evaluated the remaining 877 studies’ titles and abstracts based on the inclusion criteria. This evaluation resulted in 184 studies eligible for full-text review (Fig. 1). After screening with inclusion and exclusion criteria, 24 studies were retained, representing 3,137 participants. Two of the citations (Hattink et al., 2015; Silveira et al., 2023) each included two studies within their publication, resulting in a total of 26 comparisons included in the meta-analysis.

### Study characteristics

Descriptive statistics of study and sample characteristics can be viewed in Table 1. Study and sample characteristics and effect size data for each individual study are available on the Open Science Framework: https://osf.io/ak6ym/?view_only=f9e2d1466bf74b4c819c2038bb35ae00. The included studies were published between 2007 and 2024. 42.3% of the studies took place in North America, and 38.5% studies occurred in Europe, with 11.5% in Asia and 7.7% in Australia. Participants were on average 35.44 years old (*SD* = 12.17), 73.17% female, 18.73% racial/ethnic minorities, and 84.71% had some post-secondary education. The most common study population consisted of individuals in helping professions, such as healthcare professionals, comprising 53.85%. Other samples were recruited from the general population (30.77%), college and graduate students (11.55%), and broader university communities (3.85%). Only one study included in this meta-analysis included a sample population with a clinical diagnosis, which was Parkinson’s disease. The majority of studies used non-specific control conditions (61.54%). The digital interventions used included self-guided online modules (30.77%), VR (26.92%), smartphone apps (19.23%), video games (11.54%), and audio and/or video (11.54%). The majority of studies did not include a follow-up assessment time point (76.92%). For studies with a follow-up assessment, the average follow-up length post-treatment was 2.16 weeks (*SD* = 3.92, range = one to 12 weeks).

**Table 1:** Descriptive statistics of sample and study characteristics

Characteristics	k (% of studies)	Mean (SD)
Age		35.44 (12.17)
% Female		73.17 (19.9)
% REM		18.73 (20.72)
% Some College		84.71 (27.39)
Country		
Australia	2 (7.69%)	
Canada	1 (3.85%)	
Germany	2 (7.69%)	
Italy	1 (3.85%)	
Japan	1 (3.85%)	
Netherlands	2 (7.69%)	
Singapore	1 (3.85%)	
Spain	2 (7.69%)	
Taiwan	1 (3.85%)	
UK	1 (3.85%)	
UK and Netherlands	2 (7.69%)	
USA	10 (38.46%)	
Diagnosis		
None	25 (96.15%)	
Parkinson’s Disease	1 (3.85%)	
Career Type		
College Students	2 (7.69%)	
General Population	8 (3.08%)	
Graduate Students	1 (3.85%)	
Helper	14 (53.85%)	
University Community	1 (3.85%)	
Deliberate Practice or Feedback		
Yes	7 (26.92%)	
No	19 (73.08%)	
Control Group Type		
Non-specific	16 (61.54%)	
Active	10 (38.46%)	
Treatment Type		
Smartphone App	5 (19.23%)	
Audio and/or Video	3 (11.54%)	
Self-guided Online Modules	8 (30.77%)	
Video Game	3 (11.54%)	
Virtual Reality	7 (26.92%)	
Human-supported Intervention		
Yes	1 (3.85%)	
No	25 (96.15%)	
Treatment Weeks		4.53 (5.53)
Follow-up Length (Weeks)		2.16 (3.92)
Intention-to-treat N		146.96 (99.78)

% REM = Percentage of racial or ethnic minorities

### Effectiveness of digital interventions on empathy

**Fig. 2:**
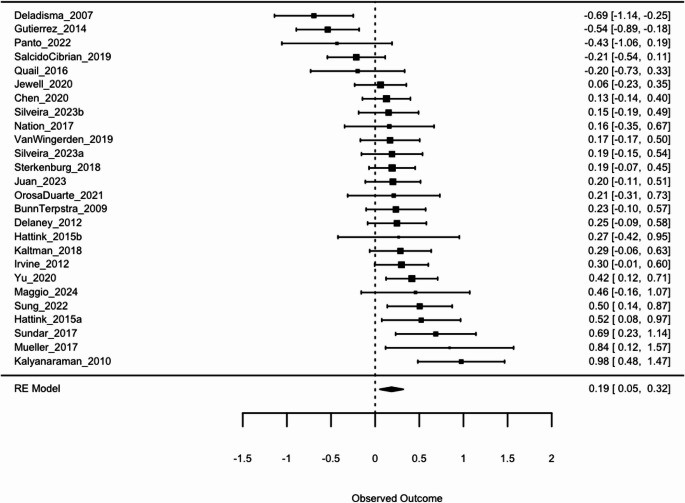
Forest Plot of Effect Sizes Across 26 Comparisons

**Fig. 3:**
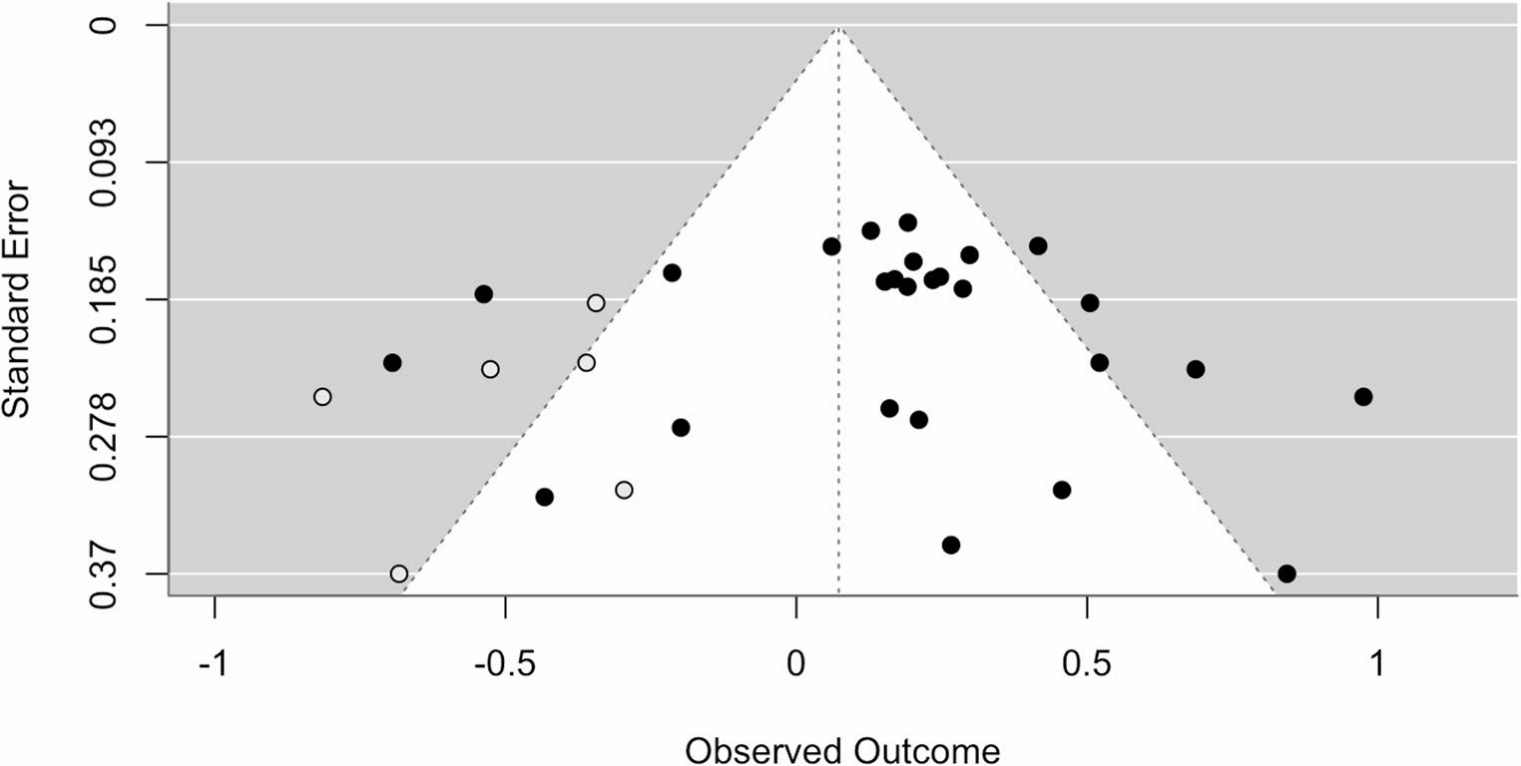
Funnel Plot Depicting Results of Trim-and-Fill Adjustment

**Fig. 4:**
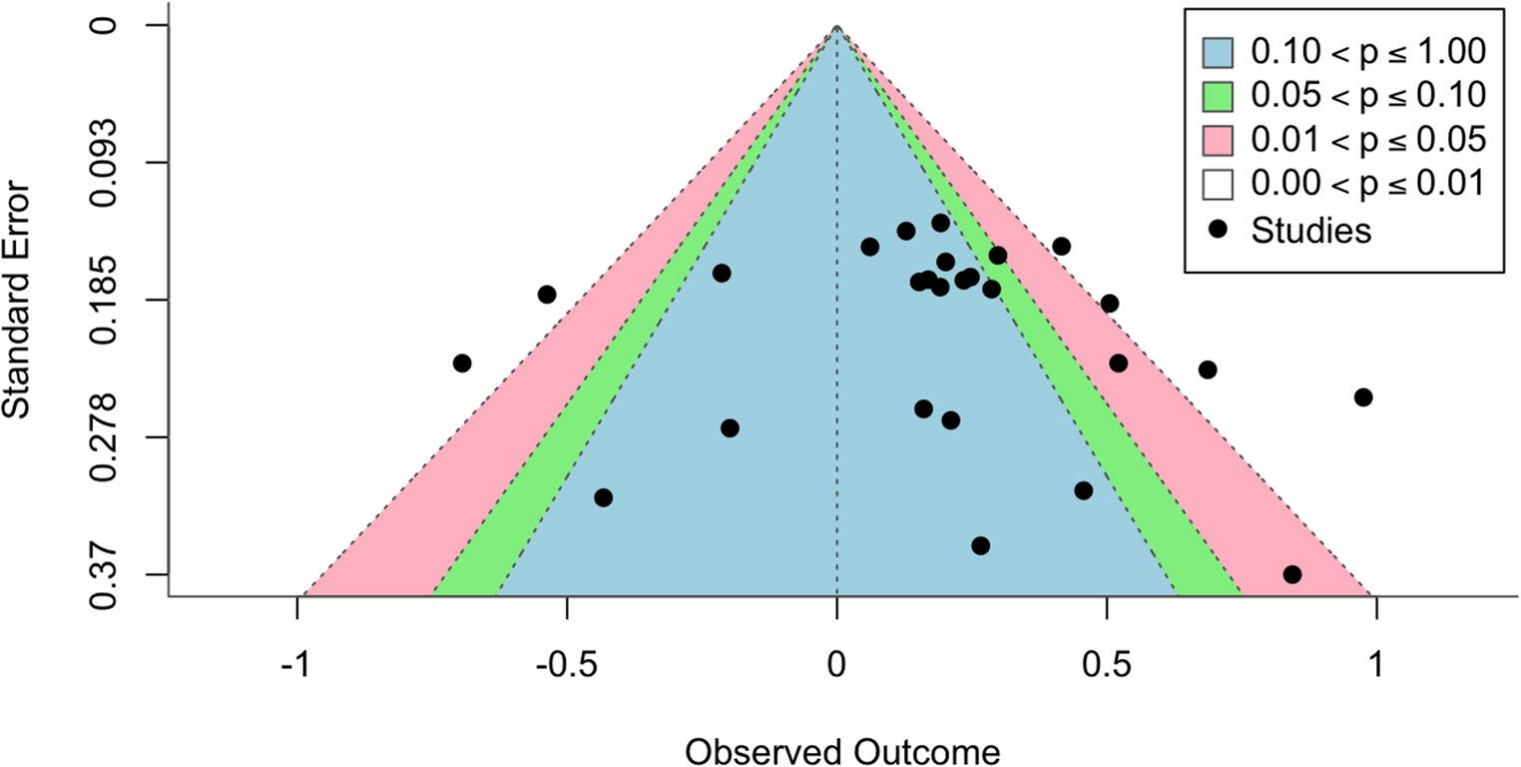
Color-Coded Enhancement Of Funnel Plot

The overall estimated effect size (*g* = 0.19, 95% CI [0.05, 0.32]) was statistically significant (*p* =.006), indicating that digital interventions have a small positive effect on empathy in adults on average. Figure 2 presents the forest plot. However, when accounting for publication bias using trim-and-fill adjustment, the estimated effect size became smaller (*g* = 0.07, 95% CI [−0.07, 0.22]) and non-significant (*p* =.322). See Fig. 3 for funnel plot depicting results of trim-and-fill adjustment and Fig. 4 for color contoured funnel plot. Specifically, the trim-and-fill analysis indicated an under-representation of studies with larger standard errors showing non-significant results. This pattern suggests that the initial positive effect size might have been inflated due to publication bias, where smaller studies with disproportionately positive findings are more likely to be published. Additionally, heterogeneity was quite high both before (67.84% [95% CI 48.33, 85.80]) and after trim-and-fill analysis (76.06%, [95% CI [63.11, 88.14]), indicating the presence of unexplained variability between studies. Six studies with follow-up data were analyzed separately and a small effect was found (*g* = 0.29, 95% CI [0.07,0.5], *p* =.008). This effect was robust to trim-and-fill analysis and no missing studies were imputed, though trim-and-fill was likely underpowered due to the small number of studies (Duval & Tweedie, 2000).

### Moderator tests

The moderators evaluated in this meta-analysis included age, diagnosis, career type, dose/treatment length, deliberate practice or feedback, control group type (i.e., active vs. non-specific, and type of digital intervention. Contrary to our hypotheses, no significant moderators were identified. See Tables 2 and 3 for moderator test results.

**Table 2:** Continuous moderator test results

Predictor	k (# of Studies)	Effect Size	Standard Error	*p*	95% CI	I^2^ [95% CI]	Q Test for Heterogeneity of Effect Sizes (QE [df] *p*)	Q Test of Moderators (QM [df] *p*)
**Age**	21	0.01	0.01	0.610	−0.01, 0.02	66.41% [41.46, 86.26]	53.53 (df = 19), *p* <.001	0.27 (df = 1), *p* =.611
**Dose (Treatment Weeks)**	25	0.01	0.01	0.230	−0.01, 0.04	69.69% [49.54, 86.51]	68.52 (df = 23), *p* <.001	1.18 (df = 1), *p* =.278

*k* = number of studies; *p* = p-value; CI = confidence interval; df = degrees of freedom

**Table 3:** Categorical moderator test results

Predictor	k (Number of Studies)	Effect Size	Standard Error	*p*	CI	I^2^ [95% CI]	Q Test for Heterogeneity of Effect Sizes (QE [df] *p*)	Q Test of Moderators (QM [df] *p*)
**Disorder ** **(Parkinson’s disease)**	26					69.01% [50.04, 86.68]	69.44 (df = 24), *p* <.001	0.43 (df = 1), *p* =.510
None (intercept)	25 (96.15%)	0.18	0.07	0.010	0.04, 0.31			
Parkinson’s Disease	1 (3.85%)	0.28	0.43	0.510	−0.56, 1.11			
**Career Type (all categories)**	26					57.53% [32.83, 85.40]	47 (df = 21), *p* =.0009	9.13 (df = 4), *p* =.060
Helper (intercept)	14 (53.85%)	0.24	0.08	0.003				
College Students	2 (7.69%)	0.04	0.25	0.861	−0.45, 0.53			
General Population	8 (30.77%)	−0.01	0.14	0.918	−0.28, 0.25			
Grad Students	1 (3.85%)	−0.78	0.3	0.009	−1.36, −0.19			
University Community	1 (3.85%)	−0.45	0.29	0.118	−1.02, 0.12			
**Career Type (helper vs. other)**	26					67.86% [47.94, 86.14]	66.59 (df = 24), *p* <.001	0.8 (df = 1), *p* =.370
Other (Intercept)	12 (46.15%)	0.12	0.1	0.246	−0.08, 0.32			
Helper	14 (53.85%)	0.12	0.14	0.372	−0.15, 0.39			
**Treatment Type**	26					67.74% [44.86, 85.81]	61.04 (df = 21), *p* <.001	4.40 (df = 4), *p* =.350
Smartphone App (intercept)	5 (19.23%)	0.22	0.16	0.172	−0.09, 0.53			
Audio and/or Video	3 (11.54%)	0.02	0.25	0.938	−0.46, 0.50			
Self-guided Online Modules	8 (30.77%)	0.019	0.2	0.927	−0.38, 0.42			
Video Game	3 (11.54%)	−0.43	0.26	0.096	−0.93, 0.08			
Virtual Reality	7 (26.92%)	0.04	0.21	0.856	−0.37, 0.45			
**Control Group Type**	26					68.15% [48.48, 86.25]	68.01 (df = 24), *p* <.001	0.56 (df = 1), *p* =.451
Non-specific Control (intercept)	16 (61.54%)	0.23	0.09	0.010	0.06, 0.40			
Specific Active Control	10 (38.46%)	−0.11	0.14	0.450	−0.38, 0.17			
**Deliberate Practice or Feedback**	26					67.86% [47.49, 85.73]	68.29 (df = 24), *p* <.001	1.25 (df = 1), *p* =.264
None (intercept)	19 (73.08%)	0.23	0.08	0.004	0.08, 0.39			
Deliberate Practice or Feedback	7 (26.92%)	−0.17	0.15	0.264	−0.47, 0.13			

*k* = number of studies; *p* = p-value; CI = confidence interval; df = degrees of freedom

### Results of additional analysis

Given that control groups are known to impact effects in psychological interventions generally (Goldberg at al., 2022; Wampold & Imel, 2015) as well as in digital interventions (Goldberg et al., 2023), we conducted an exploratory analysis examining effects for studies using non-specific control groups (*k* = 16) and active control groups (*k* = 10) separately. This analysis revealed a small positive effect for studies with non-specific control groups (*k* = 16, *g* = 0.22, 95% CI [0.12, 0.33], *p* <.001), and it was robust to publication bias. After trim-and-fill analysis where one study was imputed below the omnibus effect, the effect remained significant (*g* = 0.21, 95% CI [0.1, 0.32], *p* <.001). The effect for studies with active control groups was not significant (*g* = 0.13, 95% CI [−0.18, 0.44], *p* =.412) and remained non-significant with trim-and-fill analysis (*g* = 0.04, 95% CI [−0.29, 0.37], *p* =.801).

## Discussion

### Summary of findings

The development of digital interventions for improving the psychosocial skill of empathy is a recently emerging area of scientific interest. We examined 24 RCTs (26 comparisons; *n* = 3,137) comparing digital interventions with non-digital control conditions. Results indicated a small, positive effect of digital interventions on enhancing empathy in adults (*g* = 0.19), though once publication bias was taken into account, this result was rendered non-significant. Interestingly, when examining studies with follow-up data separately (*k* = 6), a small effect was found (*g* = 0.29) that was robust to publication bias, although trim-and-fill adjustment was likely underpowered. Additionally, a small effect was found for studies with non-specific control groups (*g* = 0.22), although this was an exploratory analysis and was not a preregistered primary analysis. These findings raise several questions regarding the relationship between digital interventions and empathy in adults - specifically, whether fostering empathy requires human support and cannot be achieved through digital means alone, and whether current technological limitations may be hindering the effectiveness of these interventions. We explore these issues in greater detail in the following sub-sections.

### Empathy may require human-supported interventions

As summarized above, the overall effect of digital interventions for empathy was statistically significant. However, when accounting for publication bias using trim-and-fill analysis, the estimated effect size became smaller and non-significant. The modest results from the current study contrast with a recent meta-analysis demonstrating the effects of various interventions in enhancing empathy, with the majority of studies focusing on traditional, non-digital approaches (*g* = 0.63; Teding van Berkhout & Malouff, 2016). Our results may indicate that empathy training is not particularly well-supported by digital platforms. Empathy skills may be distinct from the types of skills that are successfully supported by digital platforms, such as mindfulness skills (Linardon et al., 2024), symptom tracking skills for addressing mental and physical health concerns, and language learning skills. Empathy is a non-self-focused skill that requires understanding and connecting with the emotions of others (Cuff et al., 2016). Given the organic development of empathy that occurs in the context of human relationships (Davidov et al., 2013), it could be unrealistic to expect engaging with a digital platform to be an adequate surrogate for producing empathy.

Another important difference between empathy and other skills that are supported by digital interventions is that empathy may be more emotionally demanding (Zaki, 2014). Willingness to tap into the perspective and emotional experience of another, particularly if the other is going through something emotionally challenging, requires vulnerability to experiencing distress oneself. In fact, it is often the goal of empathy to join someone in their pain. Other skills such as relaxation skills for anxiety or behavioral activation for depression do not require taking on others’ pain in addition to one’s own. Given the emotional labor involved in empathy, emotion regulation skills may be an essential pre-requisite that allows one to manage personal distress while remaining connected to the other (Gerdes et al., 2010; Decety & Jackson, 2004). If an individual lacks emotion regulation skills, empathizing with others can be potentially dangerous, which is one reason many people are instinctively limited in their willingness to empathize (Ardenghi et al., 2021). Moreover, the amount of exposure one has to giving and receiving empathy in relationships throughout one’s life is associated with the strength of one’s own empathic capacity (Stern et al., 2024). This highlights another distinction between empathy skills and fitness, language, mindfulness, or mental health symptom reduction skills; the latter skills do not require a reservoir of specific emotional experiences from which the skill can grow. Digital interventions, which oftentimes have more modest effects than human-delivered interventions (e.g., Linardon et al., 2024), may simply not be potent enough to compensate for these influential developmental processes. It is possible that the development of empathy may be better supported by human-delivered interventions or a blend of digital interventions with human support, which can provide more personalized guidance, attunement to non-verbal communication, and offer genuine connection.

### Technological limitations may restrict the effectiveness of digital interventions on empathy

Conversely, the non-significant trim-and-fill adjusted effect size of the current study might indicate that digital interventions are simply limited by the current technology available. The analysis of studies with follow-up data had a small but significant effect (*g* = 0.29) as well as an exploratory analysis of studies with non-specific control groups (*g* = 0.22), both of which were robust to publication bias. While these effects are small, they suggest that digital interventions may be effective in the longer term and relative to non-specific control groups. Another meta-analysis, which focused on VR interventions specifically (Martingano et al., 2021), compared baseline empathy (pre-test or control condition) to empathy levels following a VR experience and found that VR was effective (*d* = 0.44). Although we did not find that intervention type moderated effects in the current meta-analysis, Martingano et al.’s findings and the current study’s small but significant findings for subsets of studies suggest that some digital interventions may still hold promise for enhancing empathy in adults.

As evidenced by Martingano et al.’s (2021) study, VR may be promising as an approach for enhancing empathy, although it is noteworthy that VR ultimately was no more effective than traditional interventions (Martingano et al., 2021). Research regarding VR’s effectiveness for fostering empathy is still in its early stages and findings are mixed overall (Martingano et al., 2021; Porcino et al., 2017), which is also reflected in the lack of a significant effect for VR as a treatment type in the current study’s moderator analysis. It may be important to explore alternative answers to the question of what type of technology could be more effective, to replace or pair with VR, for an empathy-focused digital intervention.

One answer proposed by psychotherapy researchers who examine interventions for psychosocial skills training is that interactive capability may be what is missing from current digital interventions (Imel et al., 2019). Machine learning technology that can process participants’ language and facial expressions and respond appropriately in real time may hold the most promise for cultivating a deeply human trait and skill such as empathy (Imel et al., 2019). Machine learning tools for psychotherapy training are in their infancy but thus far have demonstrated an impressive ability to identify meaningful verbalizations relevant to expressing empathy in the context of counseling (Kuo et al., 2023). There are, of course, rapid advances occurring in this area in recent years through the use of large language models (e.g., Hume AI, 2025). Interactive technology that can detect the subtleties and nuances of human expression may be able to mimic a real interaction and subsequently elicit more genuine empathy.

Another question that the current findings raise is whether digital interventions might be more effective if they incorporated a feedback component. Several of the studies included in this meta-analysis incorporated practice of an interaction (e.g., a medical professional practicing demonstrating empathy in a conversation with a simulated patient; Juan, 2023; Kaltman et al., 2018), however, only one study included feedback in response to the practice as part of the intervention model (Kaltman et al., 2018). In psychotherapy research, RCTs of deliberate practice (i.e., focused, goal-oriented skill repetition with feedback) interventions have been shown to improve interpersonal skills (e.g., Larsson et al., 2023). Additionally, Teding van Berkhout and Malouff (2016) found that use of behavioral skills training principles (instruction, modeling, practice, and feedback) was a moderator with slightly higher effect sizes than other studies. Feedback regarding demonstrations of empathy is likely a critical yet absent component from the interventions in the current meta-analysis. Advances in machine learning and artificial intelligence may well be able to provide the kind of feedback that is helpful for learning empathy.

### Theoretical and practical implications of the current study

Despite the modest effect sizes, our findings have important theoretical and practical implications. As discussed above, lack of interactivity and feedback features may restrict the efficacy of digital intervention on empathy. As noted above, this possibility aligns with Social Cognitive Theory (Bandura, 1986), which emphasizes that learning occurs through reciprocal interactions among individual characteristics (e.g., beliefs, expectations, and self-efficacy), behavior, and the environment, and that observing, modeling, and receiving feedback and reinforcement from the environment can facilitate the acquisition of new skills and behaviors. Social Cognitive Theory may provide a useful framework for guiding the development of future digital empathy interventions in ways that are socially persuasive.

On the practical level, given their accessibility and scalability, digital interventions may serve as useful tools for empathy training in settings where traditional, in-person approaches are not feasible, such as through online education, telehealth, or large-scale training for health professionals. Despite the promise of scalability, results highlight the need for innovation in this area. Intervention designers might consider integrating human support, tailored feedback, or more interactive features to enhance their effectiveness. In applied contexts, these findings suggest that digital tools may be most impactful when used to complement rather than replace traditional, in-person learning opportunities. As digital interventions for empathy continue to evolve, this meta-analysis can inform efforts to refine intervention design to optimize the efficacy of digital interventions for empathy.

### Limitations of the current study

One limitation inherent in the process of conducting a meta-analysis is the limited amount of published literature available to include in our examination. As there were only 24 studies that met inclusion criteria, the moderator analyses may have been underpowered to detect small effects (Hedges & Pigott, 2004). Additionally, authors did not consistently report potential moderators of interest, including mean age, which limited our ability to test them as predictors of effectiveness of treatment. Some publication bias assessments (e.g., for effects at follow up) were very likely underpowered (Duval & Tweedie, 2000).

Another limitation is that empathy continues to be inconsistently operationalized and measured across studies which makes it difficult to compare findings with confidence. Most of the included studies only used self-report measures to assess how well participants felt they had managed to take on the perspective or the emotions of another, which operationalizes empathy as primarily an internal experience without a behavioral component. Other studies focused more on participants’ ability to demonstrate empathy behaviorally, classifying empathy as a skill. Variability in how empathy is conceptualized across intervention studies is a significant limitation to empathy development research.

Finally, self-report measures, which are at higher risk of inaccuracy than objective measures due to response bias and subjectivity, were utilized exclusively by most studies included in this meta-analysis. Teding van Berkhout and Malouff (2016) found that changes in empathy tended to be larger when objective measures were used compared to self-report measures. In the current meta-analysis, there were too few studies that utilized an objective measure to conduct an exploratory moderator analysis on this characteristic.

## Conclusions and future research directions

The psychosocial trait and skill of empathy is foundational to human relationships and functioning communities (De Paul & Guibert, 2008; Gibb & Abela, 2008). As conflict around the world becomes increasingly pervasive, there is arguably a pressing need for developing this capacity at scale. Research on the effectiveness of digital interventions for enhancing empathy in adults is still in its infancy, though the evidence thus far indicates potential for digital interventions to have a modest and potentially non-specific (i.e., not superior to active control groups) positive impact. Digital interventions are worth continued investment and investigation given their scalability, accessibility, and cost-effectiveness (Linardon et al., 2025). Ideally future studies will investigate machine learning technology that allows for interactive experiences, tracking of verbal and non-verbal communication (e.g., facial expressions), as well as an ability to offer feedback. The most effective yet still scalable intervention for empathy may reveal itself to be a blend of digital and human-delivered support, considering the complex and relationship-based conditions typically required in traditional or organic contexts for empathy development. Next steps for research on digital interventions for enhancing empathy in adults will hopefully involve larger, higher-powered studies, an opportunity for deliberate practice and feedback, a combination of self-report and objective, behavioral measures of empathy, and interactive artificial intelligence components. 

## Supplementary Information

Supplementary Material 1

## Data Availability

Study data are available here: https://osf.io/ak6ym/?view_only=f9e2d1466bf74b4c819c2038bb35ae00.
