# Supplementary material for: The effectiveness of digital interventions for enhancing empathy in adults: a meta-analysis of randomized controlled trials
Source: Curr Psychol. 2026 Feb 17;45(5):523. doi: 10.1007/s12144-025-08981-8 (PMC12913337; doi:10.1007/s12144-025-08981-8)
Supplement: Supplementary file 1 [file 12144_2025_8981_MOESM1_ESM.docx]

Supplementary Information

Studies Cited in Figure 2 But Not Discussed In the Main Text

Bunn, W., & Terpstra, J. (2009). Cultivating empathy for the mentally ill using simulated auditory hallucinations. Academic Psychiatry, 33, 457-460. https://doi.org/10.1176/appi.ap.33.6.457

Chen, H. C., Ignacio, J., & Yobas, P. (2020). Evaluation of the symptom-focused health assessment and empathy program for undergraduate nursing students: A randomized controlled trial. Nurse Education Today, 94, 104566. https://doi.org/10.1016/j.nedt.2020.104566

Delaney, R., Nelson, C., Pacifici, C., White, L., & Smalley, B. K. (2012). Web-enhanced preservice training for prospective resource parents: A randomized trial of effectiveness and user satisfaction. Journal of Social Service Research, 38(4), 503-514. https://doi.org/10.1080/01488376.2012.696416

Gutierrez, B., Kaatz, A., Chu, S., Ramirez, D., Samson-Samuel, C., & Carnes, M. (2014). “Fair Play”: a videogame designed to address implicit race bias through active perspective taking. Games for Health Journal, 3(6), 371-378. https://doi.org/10.1089/g4h.2013.0071

Irvine, A., Billow, M. B., Eberhage, M. G., Seeley, J. R., McMahon, E., & Bourgeois, M. (2012). Mental illness training for licensed staff in long-term care. Issues in Mental Health Nursing, 33(3), 181-194. https://doi.org/10.3109/01612840.2011.639482

Jewell, D. E. (2020). The Use of Personal Testimonials to Enhance Gender Bias Literacy in STEM [Master’s Thesis, Purdue University].

Mueller, K., Prins, R., & de Heer, H. (2017). An online intervention increases empathy, resilience, and work engagement among physical therapy students. Journal of Allied Health, 47(3), 196-203.

Nation, J. A., Wertheim, E. H., & Worthington Jr, E. L. (2017). Evaluation of an online self‐help version of the REACH forgiveness program: Outcomes and predictors of persistence in a community sample. Journal of Clinical Psychology, 74(6), 819-838. https://doi.org/10.1002/jclp.22557

Orosa-Duarte, Á., Mediavilla, R., Muñoz-Sanjose, A., Palao, Á., Garde, J., López-Herrero, V., ... & Rodríguez-Vega, B. (2021). Mindfulness-based mobile app reduces anxiety and increases self-compassion in healthcare students: a randomised controlled trial. Medical Teacher, 43(6), 686-693. https://doi.org/10.1080/0142159X.2021.1887835

Panto, F., Saito, T., Morita, N., & Ogai, Y. (2022). Mental health care for young people using video games: a pilot RCT on the development of a new intervention method toward hikikomori and futoko. F1000Research, 11, 574. https://doi.org/10.12688/f1000research.119764.1

*Penn, D. L., Ivory, J. D., & Judge, A. (2010). The virtual doppelganger: Effects of a virtual reality simulator on perceptions of schizophrenia. The Journal of Nervous and Mental Disease, 198(6), 437-443. https://doi.org/10.1097/NMD.0b013e3181e07d66 *(previously included Kalyanaraman as first author)

Quail, M., Brundage, S. B., Spitalnick, J., Allen, P. J., & Beilby, J. (2016). Student self-reported communication skills, knowledge and confidence across standardised patient, virtual and traditional clinical learning environments. BMC Medical Education, 16, 1-12. https://doi.org/10.1186/s12909-016-0577-5

Salcido-Cibrián, L. J., Ramos, N. S., Jiménez, Ó., & Blanca, M. J. (2019). Mindfulness to regulate emotions: The Mindfulness and Emotional Intelligence Program (PINEP) and its adaptation to a virtual learning platform. Complementary Therapies in Clinical Practice, 36, 176-180. https://doi.org/10.1016/j.ctcp.2019.07.003

Sterkenburg, P. S., & Vacaru, V. S. (2018). The effectiveness of a serious game to enhance empathy for care workers for people with disabilities: a parallel randomized controlled trial. Disability and Health Journal, 11(4), 576-582. https://doi.org/10.1016/j.dhjo.2018.03.003

Sundar, S. S., Kang, J., & Oprean, D. (2017). Being there in the midst of the story: How immersive journalism affects our perceptions and cognitions. Cyberpsychology, Behavior, and Social Networking, 20(11), 672-682. https://doi.org/10.1089/cyber.2017.0271

Sung, H. C., Su, H. F., Lee, W. L., Yamakawa, M., & Wang, H. M. (2022). Effects of a dementia virtual reality‐based training with peer support for home care workers: A cluster randomized controlled trial. International Journal of Geriatric Psychiatry, 37(9). https://doi.org/10.1002/gps.5799

van Wingerden, E., Wouda, M., & Sterkenburg, P. (2019). Effectiveness of m-learning HiSense APP-ID in enhancing knowledge, empathy, and self-efficacy in caregivers of persons with intellectual disabilities: a randomized controlled trial. Health and Technology, 9(5), 893-901. https://doi.org/10.1007/s12553-019-00361-0

Yu, J., Parsons, G. S., Lancastle, D., Tonkin, E. T., & Ganesh, S. (2020). “Walking in Their Shoes”: The effects of an immersive digital story intervention on empathy in nursing students. Nursing Open, 8(5), 2813-2823. https://doi.org/10.1002/nop2.860
